# Supplementary material for: Let’s (not) get together! The role of social norms on social distancing during COVID-19
Source: PLoS One. 2021 Mar 2;16(3):e0247454. doi: 10.1371/journal.pone.0247454 (PMC7924783; doi:10.1371/journal.pone.0247454)
Supplement: S1 Fig — The figure shows an example of the ads used for recruitment. (PDF) [file pone.0247454.s001.pdf]

S1 Fig. Facebook Ads - Recruitment. The figure shows an example of the ads used for recruitment.

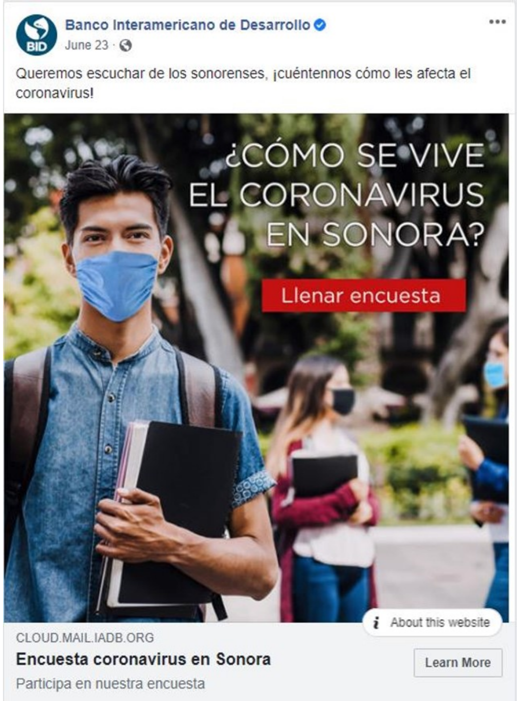

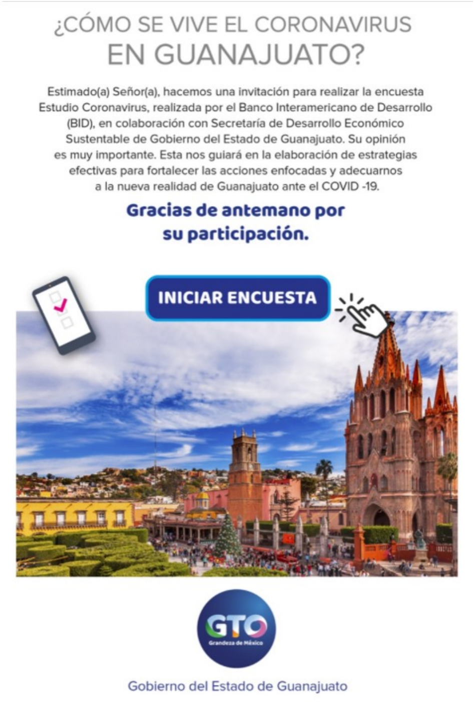

Translation of left ad	Translation of right ad
Inter-American Development Bank	How do you live with Coronavirus in Guanajuato?
We want to hear from Sonorans. Tell us how the coronavirus is affecting you!	Dear Madam[/Sir], we invite you to fill out the Coronavirus Study survey, carried out by the Inter-American Development Bank (IDB), in collaboration with the Secretariat for Sustainable Economic Development of the Government of the State of Guanajuato. Your opinion is very important. it will guide us in the development of effective strategies to strengthen focused actions and adapt to the new reality of Guanajuato in the face of COVID-19.
How is the coronavirus experienced in Sonora?	Thanks in advance for your participation.
Fill out the survey	Start survey
Sonora virus survey	Guanajuato state government
Take part in our survey	
