# Supplementary material for: Let’s (not) get together! The role of social norms on social distancing during COVID-19
Source: PLoS One. 2021 Mar 2;16(3):e0247454. doi: 10.1371/journal.pone.0247454 (PMC7924783; doi:10.1371/journal.pone.0247454)
Supplement: S1 Appendix — This document presents all the questions (in Spanish—original language of the survey—and English) used to construct dependent and treatment variables, as well as covariates. (PDF) [file pone.0247454.s002.pdf]

S1 Appendix. Survey questions. This document presents all the questions (in Spanish - original language of the survey - and English) used to construct dependent and treatment variables, as well as covariates.

**Variable**	**Values**	**Preguntas y Respuestas**	**Questions and Answers**
Welcome		¡Bienvenido/a y gracias por participar!	Welcome and thank you for participating!
		Este es un estudio llevado a cabo por el Banco Interamericano de Desarrollo (BID) y el Instituto Tecnológico Autónomo de México que nos ayudará a comprender mejor la epidemia del Coronavirus. El BID se encarga de apoyar el desarrollo de países como México, por lo que tus respuestas serán un insumo importante para formular soluciones a los retos de la pandemia.	This is a study carried out by the Inter-American Development Bank (IDB) and the Instituto Tecnologico Autonomo de Mexico that will help us better understand the Coronavirus epidemic. The IDB is responsible for supporting the development of countries like Mexico, so your responses will be an important input in formulating solutions to the challenges of the pandemic.
		El cuestionario toma 10 minutos. Todas tus respuestas son confidenciales y se utilizarán exclusivamente para propósitos de investigación científica.	The questionnaire takes 10 minutes. All your answers are confidential and will be used exclusively for scientific research purposes.
		Tu participación es voluntaria y la puedes terminar en cualquier momento y por cualquier razón. Al final del esta breve encuesta tendrás oportunidad de elegir si deseas participar en la siguiente fase del estudio.	Your participation is voluntary and can be terminated at any time and for any reason. At the end of this short survey you will have the opportunity to choose if you want to participate in the next phase of the study.
		Al hacer click sobre la flecha que aparece abajo, confirmas tener 18 o más años de edad.	By clicking on the arrow below, you confirm that you are 18 years of age or older.
		¡Muchas gracias por participar!	Thank you very much for participating!
Female		¿Cuál es tu género?	What is your gender?
	0	Femenino	Female
	1	Masculino	Male
	5	Otro	Other
	6	No sé / prefiero no responder	I don't know / I prefer not to answer
Age (group)		¿Cuál es tu edad?	How old are you?
	1	18-24	18-25
	2	25-39	25-40
	3	40-55	40-56
	4	55-64	55-65
	6	65+	65+
	7	No sé / prefiero no responder	I don't know / I prefer not to answer
Location		¿En qué estado y municipio vives?	In what state and municipality do you live in?
		[U+25BC] Estado	[U+25BC] State
		[U+25BC] Municipio	[U+25BC]Municipality
Education (group)		¿Cuál fue el último nivel educativo que completaste?	What was the highest level of education you completed?
	1	No fui a la escuela	I did not go to school
	2	Primaria	Primary
	3	Secundaria	Secondary
	4	Preparatoria	High School
	5	Superior o universitaria	Higher or university
	6	Maestría u otro nivel más avanzado	Master's degreer another more advanced level
	7	No sé / prefiero no responder	I don't know / I prefer not to answer
Older 65		Incluyéndote a ti, ¿en este momento vive en tu hogar algún adulto mayor de 65 años?	Including you, is there an adult over 65 living in your household at this time?
	1	Sí	Yes
	2	No	No
	3	No sé / prefiero no responder	I don't know / I prefer not to answer
Exposed H1N1		Durante la crisis del virus de influenza H1N1 en el verano del año 2009 en México, ¿tú o alguien que conoces se enfermaron del virus?	During the H1N1 influenza virus crisis in the summer of 2009 in Mexico, did you or someone you know become ill with the virus?
	1	Sí	Yes
	2	No	No
	3	No recuerdo / No sé	I don't remember / I don't know

Variable	Values	Preguntas y Respuestas	Questions and Answers
**Start of Block: Perceptions**			
Prob. Infection	0-100	En tu opinión, ¿qué tan probable es que tú te contagies de Coronavirus en los siguientes 6 meses?<br>Barra deslizante: variable continua<br><br>0=Nada probable<br>100= Sumamente probable<br>Barra deslizante: 0-100	In your opinion, how likely is it that you will get Coronavirus in the next 6 months?<br>Sliding bar: continuous variable<br><br>0=Not likely<br>100= Highly probable<br>Sliding bar: 0-100
Prob. Hospital	0-100	En tu opinión, si una persona de tu edad se contagia de Coronavirus, ¿qué tan probable es que termine hospitalizado/a?<br>Barra deslizante: variable continua<br><br>0=Nada probable<br>100= Sumamente probable<br>Barra deslizante: 0-100	In your opinion, if a person your age is infected with Coronavirus, how likely is it that they will end up hospitalized?<br>Sliding bar: continuous variable<br><br>0=Not likely<br>100= Highly probable<br>Sliding bar: 0-100
Exposed COVID-19	 <br>1<br>2<br>4	¿Tú o algún amigo, familiar o colega tuyo han tenido Coronavirus?<br>Sí<br>No<br>No sé / prefiero no responder	Have you or a friend, relative or colleague of yours had Coronavirus?<br>Yes<br>No<br>I don't know / I prefer not to answer
Death COVID-19	 <br>1<br>2<br>4	¿Conoces a alguien que haya muerto por Coronavirus?<br>Sí<br>No<br>No sé / prefiero no responder	Do you know someone who has died from Coronavirus?<br>Yes<br>No<br>I don't know / I prefer not to answer
**Start of Block: Risk Perception**			
Risky Inside Restaurant	 <br><br>1<br>2<br>3<br>4	Ahora piensa en el riesgo de contagio. ¿Qué tan riesgoso crees que es ir a comer a un restaurante cerrado?<br>Riesgo alto<br>Riesgo medio<br>Riesgo bajo<br>No sé / prefiero no responder	Now think about the risk of contagion. How risky do you think it is to eat at an indoor restaurant?<br><br>High risk<br>Medium risk<br>Low risk<br>I don't know / I prefer not to answer
Risky Inside Office	 <br><br>1<br>2<br>3<br>4	Ahora piensa en el riesgo de contagio. ¿Qué tan riesgoso crees que es ir a trabajar a la oficina con todos los colegas?<br>Riesgo alto<br>Riesgo medio<br>Riesgo bajo<br>No sé / prefiero no responder	Now think about the risk of contagion. How risky do you think it is to go to work at the office with all your colleagues?<br>High risk<br>Medium risk<br>Low risk<br>I don't know / I prefer not to answer
Risky Inside Gym	 <br>1<br>2<br>3<br>4	Ahora piensa en el riesgo de contagio. ¿Qué tan riesgoso crees que es ir a un gimnasio cerrado?<br>Riesgo alto<br>Riesgo medio<br>Riesgo bajo<br>No sé / prefiero no responder	Now think about the risk of contagion. How risky do you think it is to go to an indoor gym?<br>High risk<br>Medium risk<br>Low risk<br>I don't know / I prefer not to answer

Variable	Values	Preguntas y Respuestas	Questions and Answers
**Start of Block: Behavior**			
Visit others	 <br><br><br>1<br>2	En los últimos 7 días, ¿tú o alguien en tu hogar realizaron alguna de las siguientes actividades?<br><br>Asistir a una reunión o fiesta con más de 10 personas<br>Sí<br>No	In the last 7 days, did you or someone in your household perform any of the following activities?<br><br>Attend a meeting or party with more than 10 people<br>Yes<br>No
Attend Party	 <br><br>1<br>2	En los últimos 7 días, ¿tú o alguien en tu hogar realizaron alguna de las siguientes actividades?<br><br>Visitar a parientes o amigos en su casa.<br>Sí<br>No	In the last 7 days, did you or someone in your household perform any of the following activities?<br><br>Visit relatives or friends at home<br>Yes<br>No
Distance Peers	 <br><br><br>5<br>6	Pensando en tus vecinos y conocidos, ¿dirías que en general toman o no toman las siguientes medidas?<br><br>Mantener sana distancia de otras personas<br>Sí<br>No	Thinking of your neighbors and acquaintances, would you say that in general they do or do not take the following measures?<br><br>Keep distance with others<br>Yes<br>No
**Start of Block: Vignette_Mariana**			
high normative /high empirical		Piensa con cuidado en la siguiente situación hipotética:<br><br>Mariana vive en Sonora y ha venido respetando los lineamientos de salud por la epidemia de Coronavirus. Una amiga cumple años e invitó a Mariana, junto con otros 20 amigos, a asistir a una reunión dentro de su casa.<br><br>Mariana sabe que sus amigos piensan que no es debido asistir a la reunión y pocos asistirán.	Think carefully about the following hypothetical situation:<br><br>Mariana lives in Sonora and has been respecting the health guidelines for the Coronavirus epidemic. A friend has a birthday and invited Mariana, along with 20 other friends, to attend a meeting inside her home.<br><br>Mariana knows that her friends think it is not appropriate to attend the meeting and few will attend.
high normative / low empirical		Piensa con cuidado en la siguiente situación hipotética:<br><br>Mariana vive en Sonora y ha venido respetando los lineamientos de salud por la epidemia de Coronavirus. Una amiga cumple años e invitó a Mariana, junto con otros 20 amigos, a asistir a una reunión dentro de su casa.<br><br>Mariana sabe que sus amigos piensan que no es debido asistir a la reunión pero la mayoría asistirá.	Think carefully about the following hypothetical situation:<br><br>Mariana lives in Sonora and has been respecting the health guidelines for the Coronavirus epidemic. A friend has a birthday and invited Mariana, along with 20 other friends, to attend a meeting inside her home.<br><br>Mariana knows that her friends think it is not appropriate to attend the meeting but most will attend.
low normative / low empirical		Piensa con cuidado en la siguiente situación hipotética:<br><br>Mariana vive en Sonora y ha venido respetando los lineamientos de salud por la epidemia de Coronavirus. Una amiga cumple años e invitó a Mariana, junto con otros 20 amigos, a asistir a una reunión dentro de su casa.<br><br>Mariana sabe que sus amigos piensan que está bien asistir a la reunión y la mayoría asistirá.	Think carefully about the following hypothetical situation:<br><br>Mariana lives in Sonora and has been respecting the health guidelines for the Coronavirus epidemic. A friend has a birthday and invited Mariana, along with 20 other friends, to attend a meeting inside her home.<br><br>Mariana knows that her friends think it is okay to attend the meeting and most will attend.
low normative / high empirical		Piensa con cuidado en la siguiente situación hipotética:<br><br>Mariana vive en Sonora y ha venido respetando los lineamientos de salud por la epidemia de Coronavirus. Una amiga cumple años e invitó a Mariana, junto con otros 20 amigos, a asistir a una reunión dentro de su casa.<br><br>Mariana sabe que sus amigos piensan que está bien asistir a la reunión pero pocos asistirán.	Think carefully about the following hypothetical situation:<br><br>Mariana lives in Sonora and has been respecting the health guidelines for the Coronavirus epidemic. A friend has a birthday and invited Mariana, along with 20 other friends, to attend a meeting inside her home.<br><br>Mariana knows that her friends think it is okay to attend the meeting but few will attend.

Variable	Values	Preguntas y Respuestas	Questions and Answers
**Start of Block: Dependent Variables for Survey Experiment**			
Vignette Attend		¿Crees que Mariana asistirá a la reunión o no lo hará?	Do you think Mariana will attend the meeting or will she not?
	1	Si asistirá	Yes, she will attend
	2	No asistirá	No, she will not attend
	4	No sé / prefiero no responder	I don't know / I prefer not to answer
Vignette Norm		En tu opinión, ¿Mariana debería o no debería asistir a la reunión?	In your opinion, should Mariana or should she not attend the meeting?
	1	Si debería	Yes, she should attend
	2	No debería	No, she should not attend
	4	No sé / prefiero no responder	I don't know / I prefer not to answer
**Start of Block: Follow up invitation**			
Follow Up		Muchas gracias por haber completado esta encuesta. Para contribuir a entender la epidemia y reducir el contagio,<br><br>¿desearías participar en una breve encuesta de seguimiento en algunas semanas?<br><br>Como agradecimiento, el Instituto Tecnológico Autónomo de México rifará 2 teléfonos iPhone nuevos de último modelo entre las personas que completen la encuesta de seguimiento.	Thank you very much for completing this survey. To help understand the epidemic and reduce contagion,<br><br>Would you like to participate in a short follow-up survey in a few weeks?<br><br>As a thank you, the Instituto Tecnológico Autónomo de México will raffle off 2 new latest-model iPhone phones among the people who complete the follow-up survey.
	4	Si	Yes
	5	No	No
Email		Correo electrónico:	Email:
Notas		Recibirás un mensaje de confirmación. El número de WhatsApp del estudio es: 55 8015 1415. ¡Toma nota por favor!<br><br>Te recordamos que tu información de contacto solamente se usará para fines del estudio, se guardará de manera encriptada y segura, y se borrará cuando termine el estudio.	You will receive a confirmation message. The WhatsApp number of the study is: 55 8015 1415. Please take note!<br><br>We remind you that your contact information will only be used for study purposes, it will be stored in an encrypted and secure manner, and will be deleted when the study ends.
Recommend		¿Te gustaría ofrecerle la oportunidad de participar en el estudio a amigos o conocidos?<br><br>Si sí, por favor ingresa una o más direcciones de correo electrónico (opcional)	Would you like to offer friends or acquaintances the opportunity to participate in the study?<br><br>If yes, please enter one or more email addresses (optional)
Satisfaction		Por último, quisiéramos saber cómo fue tu experiencia con esta encuesta. ¿Qué tan amena te pareció la encuesta?	Finally, we would like to know how was your experience with this survey. How enjoyable did you find the survey?
	1	Muy amena	Very enjoyable
	2	Algo amena	Somewhat pleasant
	5	Ni amena ni aburrida	Neither enjoyable not boring
	3	Poco amena	Little pleasant
	4	Aburrida	Boring
	6	No sé / prefiero no responder	I don't know / I prefer not to answer
Calidad		Por último, te pedimos tu sincera opinión. ¿Nos recomendaría utilizar sus respuestas como parte del estudio? Si por alguna razón no respondiste con cuidado o no leiste las preguntas al responder, por favor selecciona "No utilizar" para evitar afectar la calidad del estudio. No habrá ninguna consecuencia de ningún tipo para ti.	Finally, we ask for your honest opinion. Would you recommend using your answers as part of the study? If for any reason you did not answer carefully or did not read the questions when answering, please select "Do not use" to avoid affecting the quality of the study. There will be no consequence of any kind for you.?
	1	Si utilizar	Yes use
	6	No utilizar	Do not use
